# A cellulose-degrading *Bacillus altitudinis* from Tibetan pigs improved the *in vitro* fermentation characteristics of wheat bran

**DOI:** 10.1016/j.csbj.2025.03.025

**Published:** 2025-03-18

**Authors:** Junhong Wang, Teng Ma, Yining Xie, Kai Li, Chengzeng Luo, Chunran Teng, Bao Yi, Liang Chen, Hongfu Zhang

**Affiliations:** State Key Laboratory of Animal Nutrition and Feeding, Institute of Animal Science, Chinese Academy of Agricultural Sciences, Beijing 100193, China

**Keywords:** Cellulose-degrading bacteria, Wheat bran, Tibetan pigs, Fermentation

## Abstract

Wheat bran, a significant cereal by-product, is widely used in animal husbandry and the food industry. Our previous study identified a correlation between fiber utilization in Tibetan pigs and their efficient hindgut fermentation capacity. In this study, the *Bacillus altitudinis* strain Z-99, capable of cellulose degradation, was identified and isolated from Tibetan pigs. The results of solid-state fermentation demonstrated a significant reduction in neutral detergent fiber (NDF), acid detergent fiber (ADF), and hemicellulose content by 12.26 % (*P* < 0.01), 1.58 % (*P* < 0.05), and 10.68 % (*P* < 0.01), respectively, while crude protein content increased by 1.90 % (*P* < 0.01). Compared to the control group, supplementation with *Bacillus altitudinis* strain Z-99 enhanced the *in vitro* fermentation characteristics of wheat bran. In particular, it significantly increased the theoretical maximum gas production (*P* < 0.05) and elevated the content of acetic acid (*P* < 0.05), butyric acid (*P* < 0.05), isobutyric acid (*P* < 0.01), valeric acid (*P* < 0.05), and total short-chain fatty acids (*P* < 0.01). Furthermore, the addition of *Bacillus altitudinis* strain Z-99 significantly increased the abundance of *Bifidobacterium* (*P* < 0.05) and *Blautia* (*P* < 0.05), while decreasing the abundance of disease-associated *Enterococcus* (*P* < 0.01) and *Actinobacillus* (*P* < 0.05). Overall, a *Bacillus altitudinis* strain Z-99 with cellulose-degrading capacity was isolated from Tibetan pigs, and its functionality was validated through solid-state fermentation and *in vitro* fermentation methods. This study provided valuable insights into the utilization of wheat bran and the exploration of cellulose-degrading bacteria.

## Graphical Abstract

**Figure:**
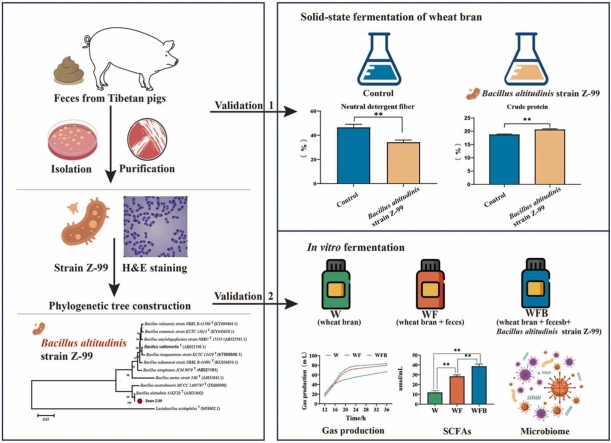

## 1 Introduction

Wheat bran, a primary by-product of wheat flour milling, has a global annual production of approximately 187 million tons. Its nutrient composition includes water, protein, fat, starch, cellulose, hemicellulose, and ash (primarily silica), and it is generally used as feed, food, or rural fuel for human and livestock [1], [2]. However, there are some limitations for wheat bran application in animal husbandry because of its high fiber content, complex properties, and poor palatability. The processing methods for high-fiber feedstuff include physical, chemical, and biological ones. Of which, the biological method is highly promising for future applications due to the advantages of mild conditions, low cost, and environmental sustainability [3].

The biological fermentation process allows wheat bran to be exposed to beneficial microorganisms that degrade complex polysaccharides into monosaccharides, thereby enhancing the utilization potential of wheat bran [4]. It has been reported that wheat bran fermented with lactic acid bacteria, affects the utilization of fiber by metabolizing conjugated phenolic compounds, disrupting their binding to cell wall polysaccharides, and thus increasing the released phenolic content [5]. Moreover, liquid fermentation of wheat bran using multiple *Bacillus* complexes significantly enhanced the dry matter fermentation rate, cellulolytic enzyme activity, and sugar production [6]. Cellulosic fermentation involves synergistic interactions among various enzymes related with cellulose degradation. The common mechanism of enzymatic cellulose hydrolysis includes the action of endonuclease β-glucanase, β-glucosidase, and exonuclease β-glucanase. Endonuclease β-glucanase randomly hydrolyzes β-1,4 glycosidic bonds along the cellulose chain, generating new chain ends. Exonuclease β-glucanase then cleaves the chain at the ends, releasing soluble disaccharides or glucose, while β-glucosidase further hydrolyzes the disaccharides into glucose, removing their inhibitory effect [7]. Cellulose-degrading bacterium, with the capacity of cellulose-degrading enzymes production, has advantages of greater environmental resistance, higher enzyme complex expression, and enhanced stability [8], becoming a hot research topic in recent years. Although fungi are well-studied for enzyme production, they are less suitable for large-scale use.

Tibetan pigs are local pig breed in the agricultural semi-agricultural and semi-pastoral areas of Tibet, western Sichuan, and northwestern Yunnan, and have developed a strong fiber utilization capacity under the natural selection of the special local geographical environment. A previous study has found a relationship between the unique herbivory of Tibetan pigs with the distinct microflora in their intestinal tract [9]. Meng et al. [10] studied the biochemical properties of cellulase produced from a *Bacillus subtilis* BY-3 strain with the capacity of degrading corn stover. But the related *in vivo* and *in vitro* verification experiments are still lacking.

Active probiotics with series of important biological functions, such as, regulating the balance of intestinal flora, resisting pathogenic bacterial infections, promoting digestion and absorption, improving feed efficiency, are increasingly vital in animal husbandry [11]. It has shown that supplementary with active probiotics enhanced energy utilization and reduced maintenance costs, thereby improving feed conversion ratio which is crucial for the development and sustainability in pig industry [12]. It is very expensive and time-consuming to conduct *in vivo* animal experiment to identify new probiotics as feed additives. Therefore, testing conducted with *in vitro* models are gaining more attention for their convenience and efficiency [13]. Ruminants, with abundant microorganisms and a unique rumen environment, can degrade cellulose into short chain fatty acids (SCFAs) and other beneficial compounds for absorption. However, for monogastric animals like pigs, they can only utilize limited cellulose by the way of hindgut fermentation. Meanwhile, *in vitro* fermentation models stimulated the hindgut of pigs are very rare. In our previous research, Gao et al. [14] developed an *in vitro* porcine hindgut fermentation model to study the utilization of high-fiber alfalfa in Tibetan pigs. Additionally, an *in vitro* batch fermentation model in humans and pigs has been used to assess the assimilation of prebiotics like oligofructose (FOS), oligogalactose (GOS), and konjac glucomannan oligosaccharide (KGMO) with the probiotic strain *Lactobacillus amyloliquefaciens* DSM 16698 [15]. Herein, we aimed to screen a new cellulose-degrading bacterial strain from Tibetan pigs and assess its potential for higher wheat bran conversion ratio with the methods of solid-state fermentation and *in vitro* fermentation. This study is crucial for better wheat bran utilization in pig breeding and for probiotic screening in Chinese local pig breeds.

## 2 Materials and methods

### 2.1 Culture media

Sodium carboxymethyl cellulose (CMC-Na) solid culture media for isolating cellulose-degrading bacteria were purchased from Shandong TuoPu Biol-engineering Co., Ltd., with the following composition: K₂PO₄ (2.5 g/L), Na₂PO₄ (g/L), CMC-Na (20 g/L), peptone (2.0 g/L), yeast extract (0.5 g/L), agar (14.0 g/L), pH 7.2 ± 0.2 at 25 °C. LB broth and solid media for bacterial cultivation were purchased from Beijing AoBoXing Bio-Technology Co., Ltd., containing peptone (10 g/L), yeast extract (5 g/L), NaCl (10 g/L), and agar (14 g/L). The cellulase-producing medium contained MgSO₄·7H₂O (0.5 g/L), K₂HPO₄ (0.5 g/L), NaCl (5 g/L), peptone (5 g/L), CMC-Na (20 g/L), CaCl₂·2H₂O (0.1 g/L), FeSO₄·7H₂O (0.1 g/L), and Tween 80 (0.1 % v/v).

### 2.2 Isolation of cellulase-degrading bacteria

Initially, one gram of fecal sample from Tibetan pigs was mixed with 99 mL sterile saline in a triangular flask and shaken (shaker CS-200, Hangzhou Yooning Instruments Co., Ltd.) at 180 r/min for 30 min to obtain a 0.01 g/mL solution. The solution was serially diluted (10⁻¹ to 10⁻⁵) with sterile saline. 100 µL of each dilution was spread onto CMC-Na solid medium and incubated at 37 °C for 24 h. Three replicates per sample and dilution were performed. After five generations of purification, single strains were spot-seeded onto fresh CMC-Na medium and incubated for 24 h. Colonies were stained with 0.1 % Congo red for 30 min, decolorized with 1 mol/mL NaCl for 30 min, and the cellulose hydrolysis zone diameters were measured. The strains were then used for cellulase activity assays: carboxymethyl cellulase (CMCase), β-glucosidase (β-Gase), exo-1,4-beta-glucanase (exoglucanase), and filter paper enzyme (FPase) activities.

Strains with cellulose-degrading capacity were inoculated into the enzyme-producing medium and incubated at 37°C, 180 r/min for 24 h. The culture was centrifuged (centrifuge CT15RE, Hitachi Koki Co., Ltd.) at 4°C, 10,000 r/min for 10 min to obtain crude enzyme extract. CMCase, β-Gase, exoglucanase and FPase activities were measured using standard methods [16], [17]. Standard glucose solutions were prepared at 0.1–0.7 mg/mL, with water as the blank control. A standard curve was generated (Supplementary Figure 1). Then, 0.1 mL of crude enzyme solution was added to assay and control tubes. In the control, the enzyme solution was inactivated by adding 1.5 mL of 3,5-dinitrosalicylic acid (DNS) reagent. In the assay tube, 0.1 mL of 1.5 mg/mL CMC-Na in acetate buffer (pH 5.5) was mixed with the enzyme solution and incubated at 50°C for 30 min. After incubation, 0.25 mL of DNS reagent was added, and both tubes were boiled for 5 min. Finally, 0.8 mL of water was added. Glucose concentration was measured at 540 nm using a microplate reader. FPase, β-Gase, and exoglucanase activities were determined similarly to CMCase, with the only difference being the substrate used: 50 mg of filter paper for FPase, 1.5 mg/mL salicylic solution for β-Gase, and 1.5 mg/mL avicel solution for exoglucanase. FPase reaction time was extended to 60 min. One unit of enzyme activity (U/mL) was defined as the amount of enzyme required to produce 1 μmol of glucose per minute from substrate hydrolysis under the specified reaction conditions.

### 2.3 Identification of cellulose-degrading bacteria

Morphological observations were performed using a Gram staining kit (Biotopped) and spore staining reagent (Solarbio). Biochemical characteristics were determined according to *Berger's Manual of Systematics of Bacteria*. Isolated strains were inoculated into LB broth (3 % concentration) with three replicates per strain. Absorbance at 600 nm was measured every 2 h, starting from 0 h, to plot the growth curve (spectrophotometer U-T5C, Yipu Instrument Manufacturing (Shanghai) Co., Ltd.). For further identification, partial 16S rDNA sequencing was performed to molecularly characterize the isolated bacterial strain. The 16S rDNA was amplified from the strain's genomic DNA using the universal primer pair 27 F/1492 R by polymerase chain reaction (PCR) method (thermocycler ABI GeneAmp® 9700, Thermo Fisher Scientific, Inc.). DNA extraction, PCR amplification, and sequencing were outsourced to Qingdao Wohai Biopharmaceutical Technology Co., Ltd. (Qingdao, China). Strain sequences were submitted to GenBank (accession: PP961918), and homologous sequences were identified using the Basic Local Alignment Search tool (BLAST). Finally, a phylogenetic tree was constructed with the Neighbor-Joining method in MEGA 11, following the method of Li et al. [18].

### 2.4 Solid-state fermentation and nutrient contents measurement

Forty grams of wheat bran along with 0.4 g of (NH₄)₂SO₄, were weighed into a triangular flask, and then sterilized at 121 °C for 15 min. Then, 20 % bacterial culture (*Bacillus altitudinis* strain Z-99) from LB broth was inoculated into the wheat bran, adjusting the liquid-to-material ratio to 1:1.5 with sterile water. An identical flask with the same water ratio served as the control (without *Bacillus altitudinis* strain Z-99). The nutrient content of the fermented materials was analyzed after 5 d of fermentation at 37°C. Crude protein was measured by the Kjeldahl method (anon, K9840), and fiber content was determined using a fully automated fiber analyzer (ALVA, F5800) with the Van Soest method. The nutrient composition of wheat bran (100 %) = moisture (10.93 %) + dry matter (DM, 89.07 %) = moisture (10.93 %) + crude protein (CP, 16.42 %) + crude fat (CF, 4.00 %) + neutral detergent fiber (NDF, 55.30 %) + nitrogen-free extractives (NFE, 8.11 %) + crude ash (5.24 %), NDF = acid detergent fiber (ADF, 14.32 %) + hemicellulose (40.98 %).

### 2.5 *In vitro* fermentation

Three groups (W, WF, and WFB) were included in the experiment of *in vitro* fermentation. The W group served as the control, with only wheat bran in the flask. For WF group, wheat bran was fermented *in vitro* with feces from Duroc Landrace Yorkshire (DLY) pigs. The WFB group designed by adding a cellulose-degrading strain (*Bacillus altitudinis* strain Z-99) to the WF group. Wheat bran was crushed and sieved through a 40-mesh filter. The bacterial inoculum was centrifuged after LB broth incubation. The *in vitro* fermentation model for the pig hindgut was previously optimized by our group. Fecal inocula were prepared according to earlier protocols [19]. Mixed feces from DLY pigs were diluted with buffer (1:5, w/v), and filtered through gauze to prepare the inoculum. Sixty milliliters of fermentation medium and 5 mL of inoculum were added to fermentation bottles, sealed, and incubated at 39°C for 36 h in an anaerobic chamber with CO₂.

### 2.6 The determination of gas production and short chain fatty acids (SCFAs)

Gas production was repeatedly recorded at 12, 14, 16, 18, 20, 24 and 36 h using an air pressure sensor (YHT, QX1208) and a syringe, and normalized according to the volatile solids (VS) content to enhance the accuracy of our assessments. The gas production was fitted in a non-linear model by SAS [14]. The model is GPt = A/(1 +(C/t)**^B^), AGPR = (A*B)/(A*C), where GPt (mL/g) is the total gas production, A (mL/g), the maximum gas volume, B, rate of increase in gas production over time, C (h), the time to reach the semi-asymptote, and AGPR (mL/g·h), the gas production when it reaches half of the maximum gas production.

After fermentation, 2 mL of the fermented liquid was transferred to a centrifuge tube and centrifuged at 10,000*g* for 10 min at 4 °C (Centrifuge CT15RE, Hitachi Koki Co., Ltd.). Next, 1.6 mL of the supernatant was transferred to a new 2 mL centrifuge tube and centrifuged at 10,000*g* for 10 min at 4 °C. The resulting supernatant was mixed with 25 % (w/v) metaphosphoric acid in a 2:8 ratio. After centrifugation at 12,000*g* for 15 min at 4 °C (Centrifuge CT15RE, Hitachi Koki Co., Ltd.), the supernatant was filtered through a 0.45-μm Milled LG filter (Millipore, Billerica, MA, USA). The SCFAs were then analyzed by gas chromatography.

### 2.7 16S rDNA sequencing and data analysis

16S rDNA sequencing was carried out by Shanghai Majorbio Bio-Pharm Technology Co., Ltd. (Shanghai, China). Briefly, 2 mL of fermentation broth was centrifuged at 10,000*g* for 10 min (centrifuge CT15RE, Hitachi Koki Co., Ltd.), and the precipitate was used for 16S rDNA second-generation sequencing and analysis. Total DNA was extracted using the MP-soil FastDNATM Spin Kit (MP Biomedicals, Southern California, USA). The V3-V4 region of the 16S rRNA gene was amplified using universal primers 338F (ACTCCTACGGGGAGGCAGCAG) and 806R (GGACTACHVGGGTWTCTAAT) with an ABI GeneAmp® 9700 thermocycler (ABI, CA, USA). After purification and quantification, PCR products were pooled in equimolar amounts and sequenced using an Illumina MiSeq sequencer.

Duplicate-free single sequences were removed from the optimized sequences. All the screened optimized sequences were then mapped to the operational taxonomic unit (OTU) representative sequences, and sequences with 97 % or greater similarity to the representative sequences were selected to generate the OTU table. Based on the OTUs, α-diversity and microbial community differences were analyzed using the *t*-test in R (version 3.3.1). Principal coordinate analysis (PCoA) was conducted using Bray-Curtis and Euclidean distances, and β-diversity was assessed through PCoA statistical analysis and visualization in R (version 3.3.1).

The correlation between SCFAs and the genera of microbial communities was analyzed using Spearman correlation analysis.

### 2.8 Statistical analysis

The experimental results are presented as mean values ± standard deviation (SD) (n = 3). Differences between groups were compared using paired *t*-tests in SAS 9.4, with significance levels of 0.01 < *P* < 0.05 and high significance at *P* < 0.01. All statistical plots were visualized using GraphPad Prism 8.

## 3 Results

### 3.1 Identification of cellulose-degrading bacteria

**Fig. 1:**
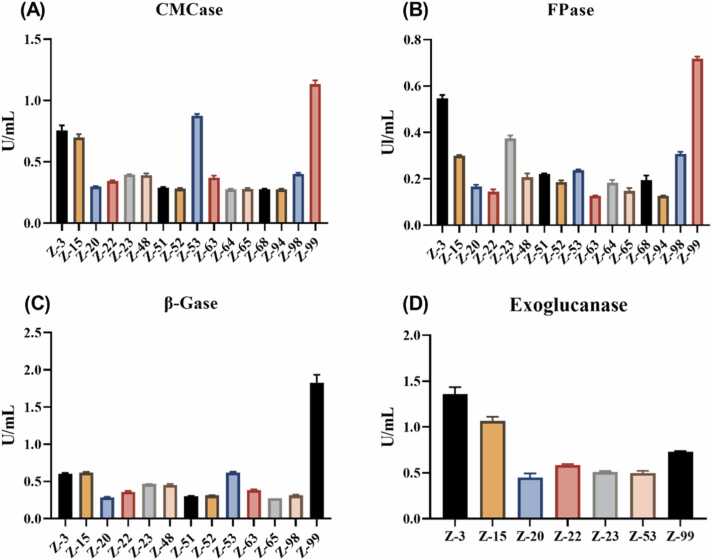
Results of cellulase activities. (A) CMCase. (B) FPase. (C) β-Gase. (D) Exoglucanase. CMCase, carboxymethyl cellulase. FPase, filter paper enzyme. β-Gase, β-glucosidase. Exoglucanase, exo-1,4-beta-glucanase.

One hundred single colonies were randomly isolated and purified on sodium carboxymethyl cellulose (CMC-Na) media. The colonies with cellulose-degrading ability were initially screened based on the ratio of cellulose hydrolysis zone diameter to the colony diameter (D/d, Supplementary Table 1). Congo red staining revealed that 79 single colonies formed hydrolysis zones. Based on the value of D/d, the top 16 colonies (Table 1) were selected for further analysis The enzyme activity results showed that all 16 strains exhibited carboxymethyl cellulase (CMCase) and filter paper enzyme (FPase) activity. Seven strains demonstrated exonuclease β-glucanase activity, and only three strains exhibited β-glucosidase (β-Gase) activity. Notably, strain Z-99 exhibited higher CMCase, β-Gase, FPase, and exoglucanase activities, with values of 1.13 U/mL, 1.83 U/mL, 0.72 U/mL, and 0.73 U/mL, respectively (Fig. 1).

**Table 1:** Diameter of cellulose hydrolysis circles.

Strain	D (mm)	d (mm)	D/d
Z−3	12.61 ± 0.46	3.58 ± 0.22	3.53 ± 0.13
Z−15	12.05 ± 0.19	3.48 ± 0.25	3.48 ± 0.26
Z−20	10.09 ± 0.13	2.51 ± 0.15	4.04 ± 0.20
Z−22	9.75 ± 0.16	2.78 ± 0.06	3.50 ± 0.09
Z−23	8.87 ± 0.32	2.45 ± 0.10	3.63 ± 0.17
Z−48	8.61 ± 0.48	2.51 ± 0.19	3.44 ± 0.10
Z−51	10.28 ± 0.65	2.92 ± 0.08	3.52 ± 0.14
Z−52	11.06 ± 0.18	3.01 ± 0.09	3.68 ± 0.06
Z−53	15.31 ± 0.16	4.49 ± 0.24	3.42 ± 0.16
Z−63	11.78 ± 1.25	3.20 ± 0.08	3.69 ± 0.48
Z−64	8.92 ± 1.01	2.51 ± 0.09	3.57 ± 0.51
Z−65	11.75 ± 0.25	3.38 ± 0.08	3.48 ± 0.15
Z−68	10.97 ± 0.53	3.20 ± 0.09	3.42 ± 0.13
Z−94	9.42 ± 1.23	2.67 ± 0.29	3.52 ± 0.08
Z−98	10.58 ± 1.60	2.65 ± 0.07	3.98 ± 0.53
Z−99	14.92 ± 0.35	4.33 ± 0.0	3.44 ± 0.03

D, the diameter of hydrolyzed circles on cellulose solid medium. d, the diameter of colonies. D/d, ratio of hydrolyzed circle diameter to colony diameter, mean ± SD, n = 3.

**Fig. 2:**
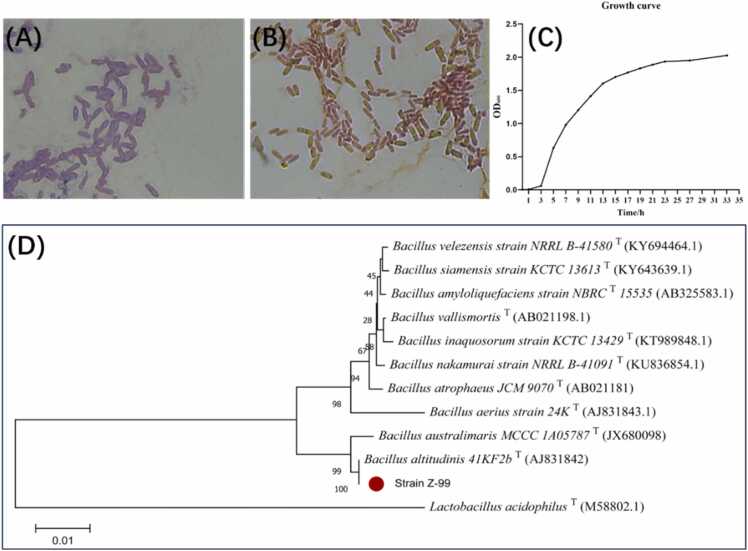
Identification of strain Z-99. (A) Gram staining. (B) Malachite green staining. (C) Growth curve. (D) Phylogenetic tree.

The colony morphology of strain Z-99 on solid medium was round, with a slightly rough, opaque, white or greyish-white surface. The Gram staining of strain Z-99 (Fig. 2A) revealed a purple color, indicating that it is a Gram-positive bacterium with a rod-like structure. Staining with malachite green showed that strain Z-99 is capable of producing spores (Fig. 2B). The biochemical characteristics of strain Z-99 were determined according to *Berger's Manual of Systematics of Bacteria* (Table 2). The growth curve of strain Z-99 (Fig. 2C) in LB liquid medium showed that the strain grew slowly during the first 3 h after inoculation as it adapted to the new nutrient environment. However, from approximately 3 h to 15 h, the strain entered a rapid growth phase, eventually reaching the plateau phase.

**Table 2:** Biochemical characteristics of strain Z-99.

Characteristics	Results	Characteristics	Results
Catalase test	**-**	Liquefaction of gelatine	**+**
Methyl red test	**-**	Casein hydrolysis assay	**+**
Indole reaction	**-**	Tyrosine hydrolysis test	**-**
D-glucose fermentation	**+**	Penylalanine deaminase test	**-**
L-Arabinose fermentation	**-**	Glucose gas production test	**-**
D-xylose fermentation	**-**	Nitrate reduction test	**+**
D-mannitol fermentation	**-**	Citrate utilization	**+**
Starch hydrolysis	**+**	Lecithinase assay	**+**

**+**, positive. **-**, negative.

The 16S rDNA sequence of strain Z-99 (1426 bp) submitted to the NCBI database and showed high similarity to *Bacillus altitudinis* (type strain, accession number: AJ831842) using the BLAST tool. The phylogenetic tree (Fig. 2D) based on the List of Prokaryotic names with Standing in Nomenclature (LPSN) revealed that strain Z-99 is closely related to *Bacillus altitudinis*. Therefore, the strain Z-99 was identified as *Bacillus altitudinis* strain Z-99 and deposited at the China General Microbiological Culture Collection Center (CGMCC) with accession number CGMCC No. 30317.

### 3.2 Effect of solid-state fermentation of *Bacillus altitudinis* strain Z-99 on wheat bran

**Fig. 3:**
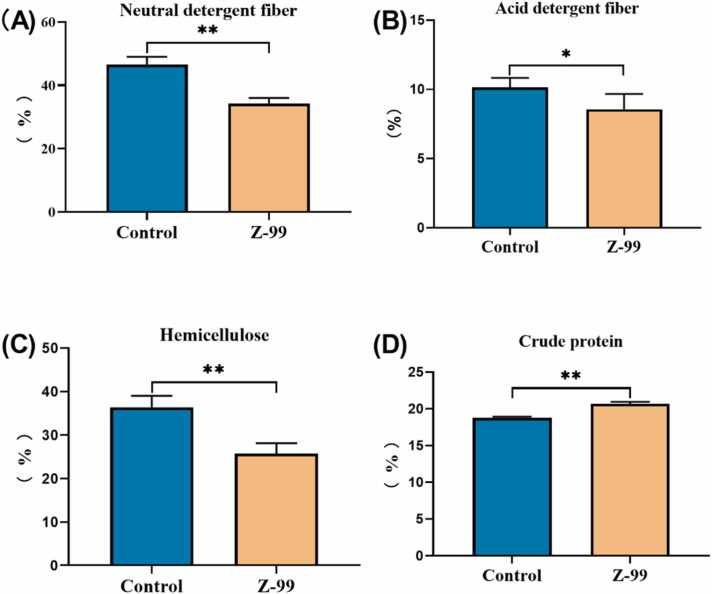
Effect of *Bacillus altitudinis* strain Z-99 on solid-state fermentation of wheat bran. (A) Neutral detergent fiber. (B) Acid detergent fiber. (C) Hemicellulose. (D) Crude protein. * 0.01 < *P* < 0.05, ** *P* < 0.01. n = 3. Z-99 represents *Bacillus altitudinis* strain Z-99.

Compared with the control group, solid-state fermentation with *Bacillus altitudinis* strain Z-99 led to a significant decrease in the neutral detergent fiber, acid detergent fiber, and hemicellulose contents of fermented wheat bran by 12.26 % (Fig. 3A, *P* < 0.01), 1.58 % (Fig. 3B, *P* < 0.05), and 10.69 % (Fig. 3C, *P* < 0.01), respectively. Meanwhile, the crude protein content was increased by 1.9 % (Fig. 3D, *P* < 0.01).

### 3.3 Effect of *Bacillus altitudinis* strain Z-99 on characteristics of wheat bran with *in vitro* fermentation

**Fig. 4:**
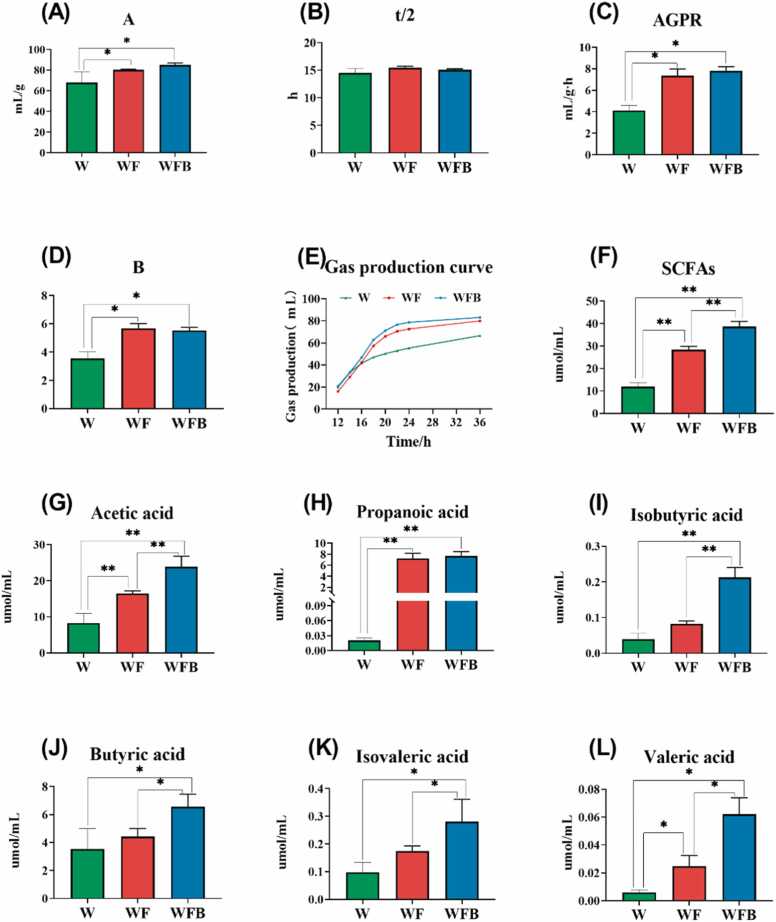
Gas and short chain fatty acids (SCFAs) production *in vitro* fermentation of wheat bran. (A) Theoretical maximum gas production. (B) The time to reach 1/2 of the maximum gas production. (C) The gas production rate reaches 1/2 of the maximum gas production. (D) The gas abundance. (E) Actual measured gas yield. (F) Total SCFAs. (G) Acetic acid. (H) Propionic acid. (I) Isobutyric acid. (J) Butyric acid. (K) Isovaleric acid. (L) Valeric acid. W, wheat bran. WF, wheat bran with feces from DLY pigs. WFB, wheat bran with feces from DLY pigs and *Bacillus altitudinis* strain Z-99. * 0.01 < *P* < 0.05, ** *P* < 0.01. n = 3.

The results showed that the parameters of *in vitro* fermentation in the WFB group were significantly affected compared to the WF group. It is worth noting that the theoretical maximum gas production (A, Fig. 4A) and the gas production rate (AGPR, Fig. 4C) when reaching half of the maximum gas production tended to be increased, while the time to reach half of the maximum gas production (t/2, Fig. 4B) and the rate of increase in gas production over time (B, Fig. 4D) were slightly decreased. As shown in Fig. 4E, the gas production was not affected with *Bacillus altitudinis* strain Z-99 supplementation (*P* > 0.05). Moreover, the content of SCFAs revealed that *Bacillus altitudinis* strain Z-99 addition significantly increased the production of total SCFAs (Fig. 4F, *P* < 0.01). Besides, the levels of acetic acid (Fig. 4G, *P* < 0.05), isobutyric acid (Fig. 4K, *P* < 0.01), butyric acid (Fig. 4J, *P* < 0.05), isovaleric acid (Fig. 4I), and valeric acid (Fig. 4L, *P* < 0.05) were significantly higher compared to the WF group, except for the content of propionic acid (Fig. 4H).

### 3.4 Effect of *Bacillus altitudinis* strain Z-99 on microbial communities with *in vitro* fermentation

16S rDNA amplicon sequencing was performed to analyze the microorganisms involved in wheat bran fermentation. Overall, a total of 267,681 valid sequences were obtained from the 6 sequenced samples after primer removal and length filtering, with 134,009 sequences from the WF group and 133,672 sequences from the WFB group. All optimized sequences were then rarefied to the minimum number of 30,793 sequences per sample and clustered into 1727 OTUs based on 97 % similarity. Additionally, the Venn diagram (Supplementary Figure 2) showed that the WF group contained 416 unique OTUs, while the WFB group contained 164 unique OTUs. The two groups shared 219 OTUs. The OTUs comprised 26 phyla, 52 classes, 105 orders, 168 families, and 295 genera. The dilution curves and sequencing coverage (> 0.98) indicated that the sequencing depth for all three groups was adequate.

**Fig. 5:**
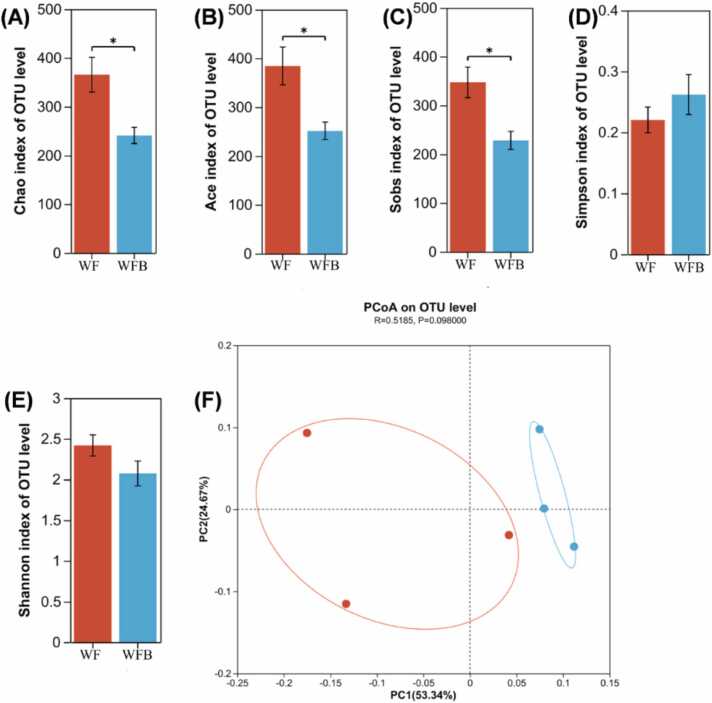
Diversity of microbial communities. (A) Chao index. (B) Ace index. (C) Sobs index. (D) Simpson index. (E) Shannon index. (F) PCoA analysis. WF, wheat bran with feces from DLY pigs. WFB, wheat bran with feces from DLY pigs and *Bacillus altitudinis* strain Z-99. *0.01 < *P* < 0.05, **P < 0.01. n = 3.

The α-diversity analysis revealed that the Ace (Fig. 5B, *P* < 0.05), Sobs (Fig. 5C, *P* < 0.05), and Chao indexes (Fig. 5A, *P* < 0.05) were significantly higher in the WF group compared to the WFB group. However, there was no significant difference in the Simpson (Fig. 5D) and Shannon indexes (Fig. 5E) between the two groups. Additionally, β-diversity analysis (Fig. 5F) showed distinct differences in microbial composition between the two groups.

**Fig. 6:**
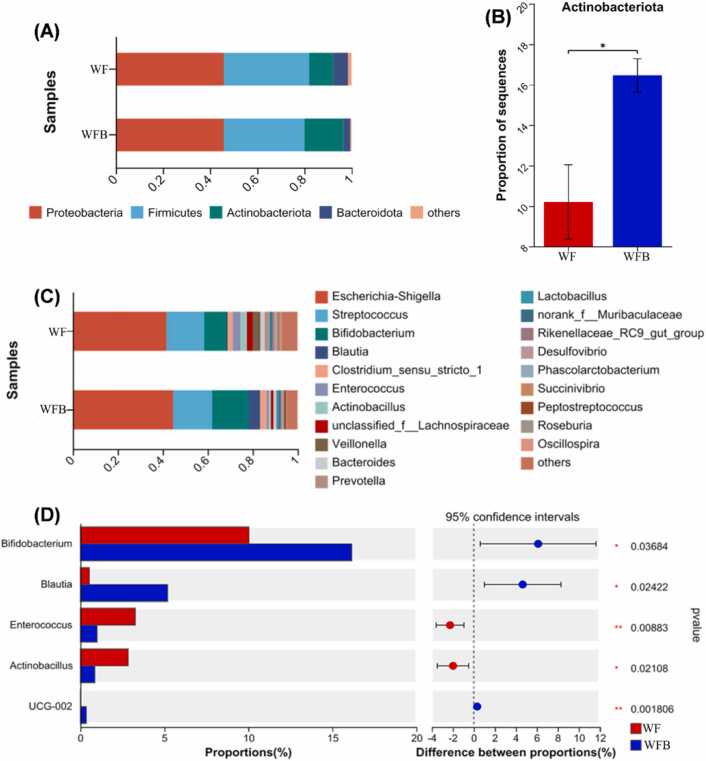
Composition of microbial communities. (A) Bar plot on phylum level. (B) Analysis of microbiota differences on phylum level. (C) Bar plot on genus level, showing the top 20 genera. (D) Analysis of microbiota differences on genus level. WF, wheat bran with feces from DLY pigs. WFB, wheat bran with feces from DLY pigs and *Bacillus altitudinis* strain Z-99. * 0.01 < *P* < 0.05, ** *P* < 0.01. n = 3.

At the phylum level, a total of 26 bacterial phyla were identified, with species exhibiting an abundance of less than 0.01 combined into the 'others' category. Firmicutes (36.18 % in WF and 34.24 % in WFB) and Proteobacteria (45.82 % in WF and 45.84 % in WFB) were the two dominant phyla in both groups, followed by Actinobacteriota (10.20 % in WF and 16.46 % in WFB) and Bacteroidota (6.24 % in WF and 2.93 % in WFB). As shown in Fig. 6A, a comparison between the WF and WFB groups revealed that the addition of *Bacillus altitudinis* strain Z-99 to wheat bran after fermentation did not affect the overall bacterial community composition at the phylum level but significantly altered the relative abundance of different phyla. Notably, supplementation with *Bacillus altitudinis* strain Z-99 to wheat bran significantly increased the relative abundance of Actinobacteriota (Fig. 6B, P < 0.05).

At the genus level (Fig. 6C), the top ten dominant genera were *Escherichia-Shigella*, *Streptococcus*, *Bifidobacterium*, *Blautia*, *Clostridium_sensu_stricto_1*, *Enterococcus*, *Enterobacter*, *unclassified_f__Lachnospiraceae*, *Veillonella*, and *Bacteroides*. The top 5 genera in terms of abundance (Fig. 6D) were then analyzed for differences, and the results showed that the addition of *Bacillus altitudinis* strain Z-99 to wheat bran significantly increased the relative abundance of *Bifidobacterium* (*P* < 0.05), *Blautia* (*P* < 0.01), and *UCG-002* (Uncultured Group, including *Ruminococcaceae UCG-002*, etc. *P* < 0.05), while greatly decreased the relative abundance of *Enterococcus* (*P* < 0.05) and *Actinobacillus* (*P* < 0.01).

### 3.5 Correlation analysis

**Fig. 7:**
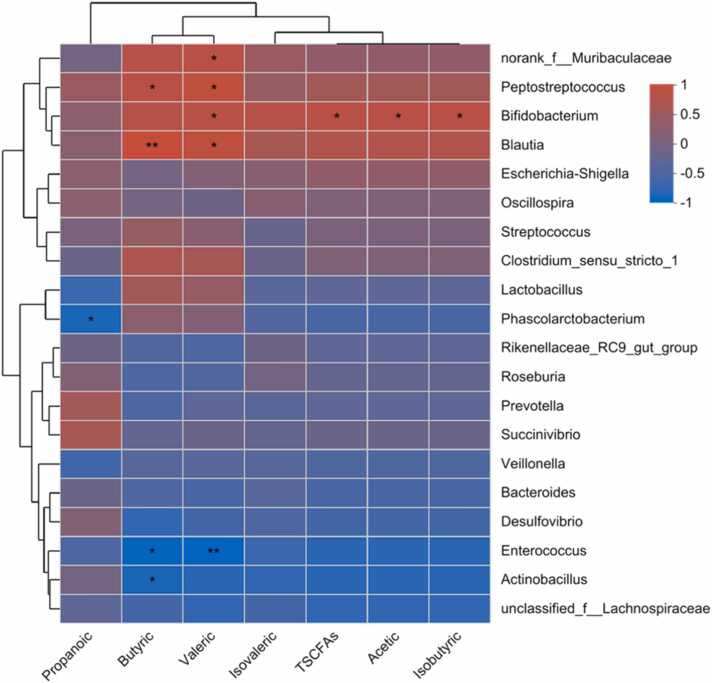
Correlation analysis of microbial genus with short chain fatty acids (SCFAs). *0.01 < *P* < 0.05, ** *P* < 0.01. n = 3.

Spearman's correlation heatmap (Fig. 7) revealed that *Bifidobacterium* was positively correlated with the production of total SCFAs (*P* < 0.05), valeric acid (*P* < 0.05), acetic acid, and isobutyric acid (*P* < 0.05). Meanwhile, *Blautia* and *Peptostreptococcus* were positively correlated with butyric acid (P < 0.01) and valeric acid (P < 0.05) production. In addition, *norank_f__Muribaculaceae* was positively correlated only with valeric acid (*P* < 0.05) production. However, *Enterococcus* was negatively correlated with butyric acid (*P* < 0.05) and valeric acid (*P* < 0.01) production, while *Actinobacillus* and *Phascolarctobacterium* were negatively correlated with butyric acid (*P* < 0.05) and propionic acid (*P* < 0.05), respectively.

## 4 Discussion

Sodium carboxymethyl cellulose (CMC-Na) as the sole carbon source in selective media plates, combined with Congo red staining, is commonly used for preliminary screening of cellulose-degrading bacteria due to its simplicity and clarity [20]. In this study, sixteen strains with cellulose-degrading ability were screened. Although Congo red can indicate cellulase activity, the weak correlation between hydrolysis zone diameter and enzyme activity makes it difficult to accurately quantify the cellulose-degrading capacity [21]. Therefore, to further qualify the cellulose-degrading capacity, it is necessary to measure cellulase activities including carboxymethyl cellulase (CMCase), β-glucosidase (β-Gase), exo-1,4-β-D-glucanase (exoglucanase), and FPase. It has reported that CMCase and β-Gase are the primary cellulase-degrading enzymes. FPase exerts the synergistic effects with other hydrolytic enzymes [22], [23]. In this study, there were 16 strains were identified with efficient cellulose degradation capacity. Of which, strain Z-99 was selected as the research target because of its general performance in cellulase secretion. Although it reported that cellulose-degrading strains, including *Bacillus pseudomycoides*, *Bacillus amyloliquefaciens*, and *Bacillus tequilensis*, have been isolated from different environmental conditions, here, the results showed that strain Z-99 had higher cellulase activity (24 h) than the activities of the prior reported strains [24], [25], [26].

Considering the shortages of morphological observations combined with biochemical tests in bacteria identification, 16S rDNA sequencing technology is used for bacterial classification and identification by comparing sequences to reference databases accurately and rapidly [27]. In this study, along with morphological and biochemical characterizations, 16S rDNA sequencing results further confirmed the strain Z-99 as *Bacillus altitudinis* with a new name *Bacillus altitudinis* strain Z-99, which was conserved in the Chinese Microbiology Conservation Centre (CMCC) subsequently. Bacterial growth curves are essential representations for characterizing bacteria metabolism and growth [28]. In this study, the growth curve of *Bacillus altitudinis* strain Z-99 was generated and depicted, which was a useful reference for its passaging and amplification in the future research and application.

To evaluate the fiber-degrading capability of *Bacillus altitudinis* strain Z-99, solid-state fermentation method was used [29]. The fermentation data showed that, compared to the control group, *Bacillus altitudinis* strain Z-99 significantly altered nutrient composition of wheat bran by reducing fiber content and increasing crude protein levels. Consistent with previous studies, the increased content of crude protein resulted from the breakdown of fiber polysaccharide by *Bacillus altitudinis* strain Z-99, leading to bacterial protein synthesis [30]. What’s more, fermentation of wheat bran increased released bioactive substances, such as, total phenols, ferulic acid, and folic acid, leading to the changes of physicochemical properties, and antioxidant activity of wheat bran. Future research will explore the effects of *Bacillus altitudinis* strain Z-99 on the structure, bioactivity, and bioavailability of wheat bran [31], [32], which is also our concern. This study provides a preliminary evaluation of a particular bacterial modifications in fiber composition alterations of wheat bran. *Bacillus altitudinis* strain Z-99 shows significant application potential for developing fibrous feedstuffs with complex physical properties.

The *in vitro* static batch fermentation model provides a novel approach for evaluating fiber-rich feed ingredients [33]. In this study, fiber-degrading *Bacillus altitudinis* strain Z-99 from Tibetan pigs was used in wheat bran fermentation to assess its potential in nutrient utilization enhancement. The results indicated that *Bacillus altitudinis* strain Z-99 improved the *in vitro* fermentation characteristics of wheat bran. Compared to the WF group, it significantly increased the theoretical maximum gas production and also increased the gas production rate at half of the maximum output. The gas production kinetics and short-chain fatty acids (SCFAs) are key indicators of *in vitro* fermentation [34]. In the present study, no significant differences were observed in fermentation parameters, except for theoretical maximum gas production. For carbohydrate fermentation in digestive tract, SCFAs (mostly acetate, propionate, and butyrate) are important substrates as energy supply for epithelial cells, and resist pathogens intrusion [35], [36], [37]. In this study, the significantly increased levels of acetic acid, butyric acid, isobutyric acid, valeric acid and total SCFAs indicated the beneficial effects of *Bacillus altitudinis* strain Z-99 as probiotics. To exclude the influence of naturally occurring microorganisms in the wheat bran itself, a blank control group (W) was designed.

16S rDNA amplicon sequencing showed that *Bacillus altitudinis* strain Z-99 supplementation affected the abundance of microbial community. Due to the competitive effects between exogenous microorganism and native microbes, the microbial diversity was not affected [38]. For the blank control group, the extremely lower microbial abundance represents weak fermentation, also validated the reliability of WF (wheat bran fermented with feces from DLY pigs) and WFB (wheat bran fermented with feces from DLY pigs and *Bacillus altitudinis* strain Z-99) groups. Consistent with previous studies, the dominant phyla in the WF and WFB groups were Firmicutes, Bacteroidota, Proteobacteria, and Actinobacteria, suggesting that Firmicutes and Proteobacteria were the dominant bacteria (more than 90 %) in the intestine of pigs [39], [40]. Supplementary with *Bacillus altitudinis* strain Z-99 increased the abundance of Actinobacteriota compared to the WF group. Actinobacteriota, a Gram-positive bacterium, includes three anaerobic families (Bifidobacteria, Propionibacteria, Corynebacteria) and one aerobic family (Streptomyces) [41]. Recently, the function of bacteria in Actinobacteria phylum was involved in intestinal barriers, material transfer, nutrient metabolism, immune function, and the brain-gut axis, indicating the modulation effects of *Bacillus altitudinis* strain Z-99 on microbiota and gut health. Moreover, *Bacillus altitudinis* strain Z-99 addition increased the abundance of beneficial *Bifidobacterium*, *Blautia*, and *UCG-002* (Uncultured Group, including *Ruminococcaceae UCG-002* et al.), while significantly reducing the abundance of *Enterococcus* and *Actinobacillus* compared to the WF group.

The correlation analysis revealed that *Bifidobacterium* and *Blautia* were positively correlated with SCFAs. Meanwhile, there was a negative correlation between *Enterococcus*, *Actinobacillus* and SCFAs. Previous studies have suggested that *Bifidobacterium* can hydrolyze glycosidic bonds using glycosyl hydrolases and cooperatively catabolize plant-derived carbohydrates like starch, FOS, GOS, XOS, inulin, and arabinoxylan [42], [43]. *Bifidobacterium* is positively linked with SCFAs production, particularly through starch and polysaccharides catabolism. It also has a significant lactic acid-producing capacity, which is utilized by "lactate utilizers" to produce butyric acid. Furthermore, *Bifidobacterium* has been shown to modulate the production of acetate and butyrate, which play a crucial role in alleviating colonic inflammation [44], [45]. *Blautia*, an anaerobic probiotic genus, secretes enzymes like β-glucosidases and O-glycosidases, crucial for polyphenol glycosylation and lignin degradation, catalyzes flavonoid transformations via demethylation, dehydroxylation, O- and C-deglycosylation, and C-ring cleavage [46]. Thus, the increased fermentation activity and SCFAs production with *Bacillus altitudinis* strain Z-99 supplementation may be due to the higher abundances of *Bifidobacterium* and *Blautia*, which enhanced the catabolism of non-starch polysaccharides in wheat bran. On the other hand, *Actinobacillus* and *Enterococcus* are harmful Gram-negative bacterium, which are associated with diseases and infections, such as, pneumonia, septicemia and urinary tract infections [47], [48], [49], [50], [51]. Therefore, the significantly reduced abundances of *Actinobacillus* and *Enterococcus* suggested the potential probiotic properties of the Tibetan pig-derived *Bacillus altitudinis* strain Z-99. Overall, *Bacillus altitudinis* strain Z-99 enhanced high-fiber wheat bran conversion to short chain of fatty acids in the hindgut and promoted intestinal health by improving microbiota composition.

There are still some limitations in this study. Firstly, *in vitro* results may not fully reflect *in vivo* conditions. Secondly, besides wheat bran, more fiber-rich feedstuff needs to be tested. Lastly, long-term detections of *Bacillus altitudinis* strain Z-99 on SCFAs bioavailability and gut health are needed. Additionally, further advanced genomic analyses would strengthen the findings and provide new insights into the utilization of *Bacillus altitudinis* strain Z-99 as feed additive candidate.

## 5 Conclusion

In summary, a cellulose-degrading strain, *Bacillus altitudinis* strain Z-99, was isolated and identified from Tibetan pigs through morphological, biochemical, and combined with 16S rDNA sequencing examinations. This strain exhibits excellent fiber degradation ability validated by solid-state fermentation on wheat bran. Further, this strain enhanced wheat bran fermentation by increasing SCFAs production and modulating microbial communities proved by an *in vitro* fermentation method. This study provides a solid foundation for cellulose-degrading bacteria screening and for feed additive development in the future.

## CRediT authorship contribution statement

**Junhong Wang:** Writing – original draft, Methodology, Investigation, Data curation. **Teng Ma:** Resources, Project administration, Funding acquisition. **Yining Xie:** Software, Formal analysis. **Liang Chen:** Resources, Project administration. **Hongfu Zhang:** Supervision, Conceptualization. **Kai Li:** Methodology, Formal analysis. **Chengzeng Luo:** Software, Resources. **Chunran Teng:** Software, Methodology. **Bao Yi:** Writing – review & editing, Validation, Supervision.

## Declaration of Competing Interest

This manuscript has been reviewed and approved by all authors. There are no commercial or financial relationships that would lead to a potential conflict of interest.

## Data Availability

All data supporting the findings in this study are presented. The 16S rDNA sequencing data have been uploaded to the GenBank database (Accession number: PP961918), and the second-generation 16S DNA sequencing data is available on NCBI's SRA with accession number PRJNA1149529.

## References

[1] Li Y., Wang H., Wang L., Qiu J., Li Z. (2023). Milling of wheat bran: influence on digestibility, hydrolysis and nutritional properties of bran protein during *in vitro* digestion. Food Chem.

[2] Apprich S., Tirpanalan Ö., Hell J., Reisinger M., Böhmdorfer S. (2014). Wheat bran-based biorefinery 2: valorization of products. LWT-Food Sci Technol.

[3] Chu X., Awasthi M.K., Liu Y., Cheng Q., Qu J. (2021). Studies on the degradation of corn straw by combined bacterial cultures. Bioresour Technol.

[4] Onipe O.O., Jideani A.I.O., Beswa D. (2015). Composition and functionality of wheat bran and its application in some cereal food products. Int J Food Sci Technol.

[5] Zhao H.M., Guo X.N., Zhu K.X. (2017). Impact of solid-state fermentation on nutritional, physical and flavor properties of wheat bran. Food Chem.

[6] Vu V., Farkas C., Riyad O., Bujna E., Kilin A. (2022). Enhancement of the enzymatic hydrolysis efficiency of wheat bran using the *Bacillus* strains and their consortium. Bioresour Technol.

[7] Percival Zhang Y.H., Himmel M.E., Mielenz J.R. (2006). Outlook for cellulase improvement: screening and selection strategies. Biotechnol Adv.

[8] Shang Z., Liu S., Duan Y., Bao C., Wang J. (2022). Complete genome sequencing and investigation on the fiber-degrading potential of *Bacillus amyloliquefaciens* strain TL106 from the Tibetan pig. BMC Microbiol.

[9] Zhao F., Yang L., Zhang T., Zhuang D., Wu Q. (2023). Gut microbiome signatures of extreme environment adaption in Tibetan pig. NPJ Biofilms Microb.

[10] Meng F., Ma L., Ji S., Yang W., Cao B. (2014). Isolation and characterization of *Bacillus subtilis* strain BY-3, a thermophilic and efficient cellulase-producing bacterium on untreated plant biomass. Lett Appl Microbiol.

[11] Yadav M.K., Kumari I., Singh B., Sharma K.K., Tiwari S.K. (2022). Probiotics, prebiotics and synbiotics: safe options for next-generation therapeutics. Appl Microbiol Biotechnol.

[12] Rao Z.X., Tokach M.D., Woodworth J.C., DeRouchey J.M., Goodband R.D. (2023). Effects of various feed additives on finishing pig growth performance and carcass characteristics: a review. Animals.

[13] Doron S., Snydman D.R. (2015). Risk and safety of probiotics. Clin Infect Dis.

[14] Gao Q., Sun G., Duan J., Luo C., Yangji C. (2022). Alterations in gut microbiota improve SCFA production and fiber utilization in Tibetan pigs fed alfalfa diet. Front Microbiol.

[15] Cardarelli H.R., Martinez R.C., Albrecht S., Schols H., Franco B.D. (2016). *In vitro* fermentation of prebiotic carbohydrates by intestinal microbiota in the presence of *Lactobacillus amylovorus* DSM 16998. Benef Microbes.

[16] Liao W., Liu S., Dong R., Xie J., Chen Y. (2022). Mixed solid-state fermentation for releasing bound polyphenols from insoluble dietary fiber in carrots *via Trichoderma viride* and *Aspergillus niger*. Food Funct.

[17] de Albuquerque Lima T., de Queiroz Baptista N.M., de Oliveira A.P.S., da Silva P.A., de Gusmão N.B. (2021). Insecticidal activity of a chemotype VI essential oil from Lippia alba leaves collected at Caatinga and the major compound (1,8-cineole) against Nasutitermes corniger and Sitophilus zeamais. Pest Biochem Physiol.

[18] Li F., Xie Y., Gao X., Shan M., Sun C. (2020). Screening of cellulose degradation bacteria from Min pigs and optimization of its cellulase production. Electron J Biotechnol.

[19] Gao Q., Li K., Zhong R., Long C., Liu L. (2021). Supplementing glycerol to inoculum induces changes in pH, SCFA profiles, and microbiota composition in *in-vitro* batch fermentation. Fermentation.

[20] Kasana R.C., Salwan R., Dhar H., Dutt S., Gulati A. (2008). A rapid and easy method for the detection of microbial cellulases on agar plates using gram's iodine. Curr Microbiol.

[21] Maki M., Leung K.T., Qin W. (2009). The prospects of cellulase-producing bacteria for the bioconversion of lignocellulosic biomass. Int J Biol Sci.

[22] Li W., Zhang W.W., Yang M.M., Chen Y.L. (2008). Cloning of the thermostable cellulase gene from newly isolated *Bacillus subtilis* and its expression in *Escherichia coli*. Mol Biotechnol.

[23] Si J., Yang C., Ma W., Chen Y., Xie J. (2022). Screen of high efficiency cellulose degrading strains and effects on tea residues dietary fiber modification: structural properties and adsorption capacities. Int J Biol Macromol.

[24] Pramanik S.K., Mahmud S., Paul G.K., Jabin T., Naher K. (2021). Fermentation optimization of cellulase production from sugarcane bagasse by *Bacillus pseudomycoides* and molecular modeling study of cellulase. Curr Res Microb Sci.

[25] Singh S., Moholkar V.S., Goyal A. (2013). Isolation, identification, and characterization of a cellulolytic *Bacillus amyloliquefaciens* strain SS35 from rhinoceros dung. ISRN Microbiol.

[26] Malik W.A., Javed S. (2024). Enhancement of cellulase production by cellulolytic bacteria SB125 in submerged fermentation medium and biochemical characterization of the enzyme. Int J Biol Macromol.

[27] Větrovský T., Baldrian P. (2013). The variability of the 16S rRNA gene in bacterial genomes and its consequences for bacterial community analyses. PLoS One.

[28] Wang W.K., Li W.J., Wu Q.C., Wang Y.L., Li S.L. (2021). Isolation and identification of a rumen *Lactobacillus* bacteria and its degradation potential of gossypol in cottonseed meal during solid-state fermentation. Microorganisms.

[29] Zhao J., Dong Z., Li J., Chen L., Bai Y. (2018). Ensiling as pretreatment of rice straw: the effect of hemicellulase and *Lactobacillus* plantarum on hemicellulose degradation and cellulose conversion. Bioresour Technol.

[30] Wang Z., Tang H., Liu G., Gong H., Li Y. (2023). Compound probiotics producing cellulase could replace cellulase preparations during solid-state fermentation of millet bran. Bioresour Technol.

[31] Tanasković S.J., Šekuljica N., Jovanović J., Gazikalović I., Grbavčić S. (2021). Upgrading of valuable food component contents and anti-nutritional factors depletion by solid-state fermentation: a way to valorize wheat bran for nutrition. J Cereal Sci.

[32] Ye G., Wu Y., Wang L., Tan B., Shen W. (2021). Comparison of six modification methods on the chemical composition, functional properties and antioxidant capacity of wheat bran. LWT-Food Sci Technol.

[33] McBurney M.I., Sauer W.C. (1993). Fiber and large bowel energy absorption: validation of the integrated ileostomy-fermentation model using pigs. J Nutr.

[34] Huang Z., Urriola P.E., Salfer I.J., Stern M.D., Shurson G.C. (2017). Differences in *in vitro* hydrolysis and fermentation among and within high-fiber ingredients using a modified three-step procedure in growing pigs. J Anim Sci.

[35] Purchiaroni F., Tortora A., Gabrielli M., Bertucci F., Gigante G. (2013). The role of intestinal microbiota and the immune system. Eur Rev Med Pharmacol Sci.

[36] Scaldaferri F., Pizzoferrato M., Gerardi V., Lopetuso L., Gasbarrini A. (2012). The gut barrier: new acquisitions and therapeutic approaches. J Clin Gastroenterol.

[37] Williams B.A., Voigt C., Verstegen M.W.A. (2017). The faecal microbial population can be representative of large intestinal microfloral activity. Proc Br Soc Anim Sci.

[38] Bai N., Zhang H., He Y., Zhang J., Zheng X. (2022). Effects of *Bacillus subtilis* A-5 and its fermented γ-polyglutamic acid on the rhizosphere bacterial community of Chinese cabbage. Front Microbiol.

[39] Arumugam M., Raes J., Pelletier E., Le Paslier D., Yamada T. (2011). Enterotypes of the human gut microbiome. Nature.

[40] Long C.X., Wu J.Q., Tan Z.J., Wang S.P. (2022). Different intestinal microbiota with growth stages of three-breed hybrid Pig. Biomed Res Int.

[41] Belizário J.E., Napolitano M. (2015). Human microbiomes and their roles in dysbiosis, common diseases, and novel therapeutic approaches. Front Microbiol.

[42] Pokusaeva K., Fitzgerald G.F., van Sinderen D. (2011). Carbohydrate metabolism in *Bifidobacteria*. Genes Nutr.

[43] Milani C., Lugli G.A., Duranti S., Turroni F., Mancabelli L. (2015). *Bifidobacteria* exhibit social behavior through carbohydrate resource sharing in the gut. Sci Rep.

[44] Nan X., Zhao W., Liu W.H., Li Y., Li N. (2023). *Bifidobacterium animalis subsp. lactis* BL-99 ameliorates colitis-related lung injury in mice by modulating short-chain fatty acid production and inflammatory monocytes/macrophages. Food Funct.

[45] Markowiak-Kopeć P., Śliżewska K. (2020). The effect of probiotics on the production of short-chain fatty acids by human intestinal microbiome. Nutrients.

[46] Liu X., Mao B., Gu J., Wu J., Cui S. (2021). *Blautia*-a new functional genus with potential probiotic properties?. Gut Microb.

[47] Zambon J.J. (1985). *Actinobacillus actinomycetemcomitans* in human periodontal disease. J Clin Periodontol.

[48] Rycroft A.N., Garside L.H. (2000). *Actinobacillus* species and their role in animal disease. Vet J.

[49] García-Solache M., Rice L.B. (2019). The *Enterococcus*: a model of adaptability to its environment. Clin Microbiol Rev.

[50] Fiore E., Van Tyne D., Gilmore M.S. (2019). Pathogenicity of *Enterococci*. Microbiol Spectr.

[51] Zaheer R., Cook S.R., Barbieri R., Goji N., Cameron A. (2020). Surveillance of *Enterococcus spp.* reveals distinct species and antimicrobial resistance diversity across a One-Health continuum. Sci Rep.

