# Supplementary material for: A cellulose-degrading Bacillus altitudinis from Tibetan pigs improved the in vitro fermentation characteristics of wheat bran
Source: Comput Struct Biotechnol J. 2025 Mar 18;27:1233–43. doi: 10.1016/j.csbj.2025.03.025 (PMC11984536; doi:10.1016/j.csbj.2025.03.025)
Supplement: Supplementary file 1 — Supplementary material [file mmc1.docx]

# **Supplementary Figures**

**Supplementary Figure 1**


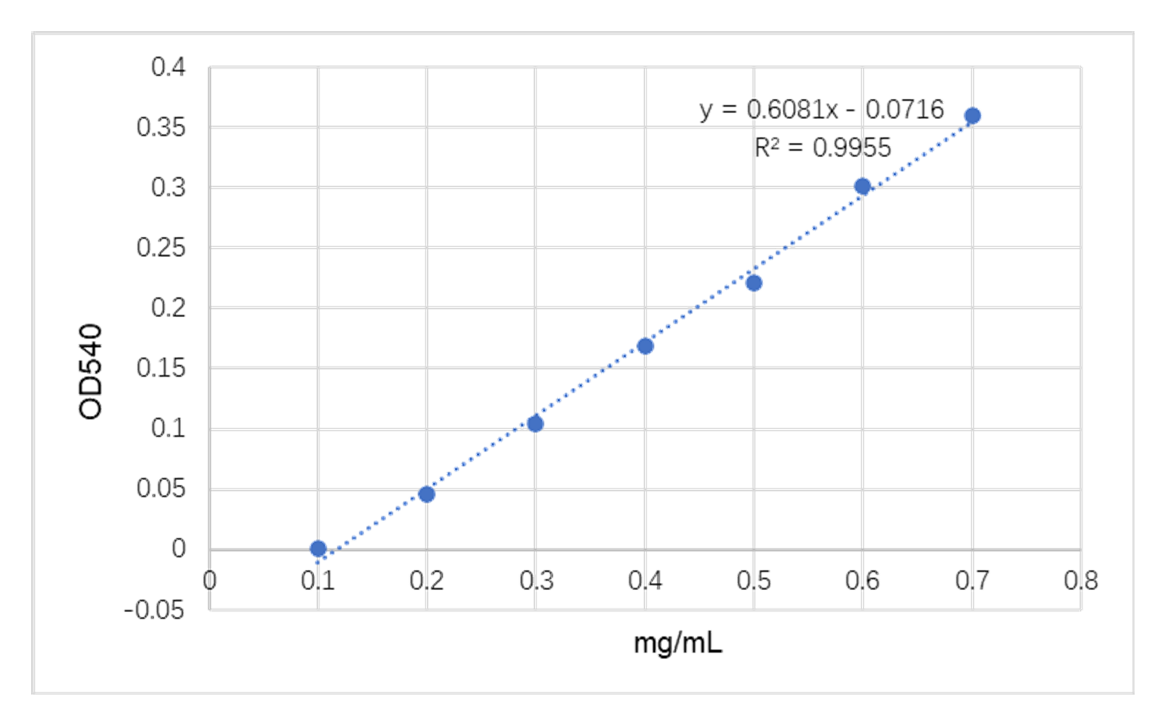


**Supplementary Figure 2**


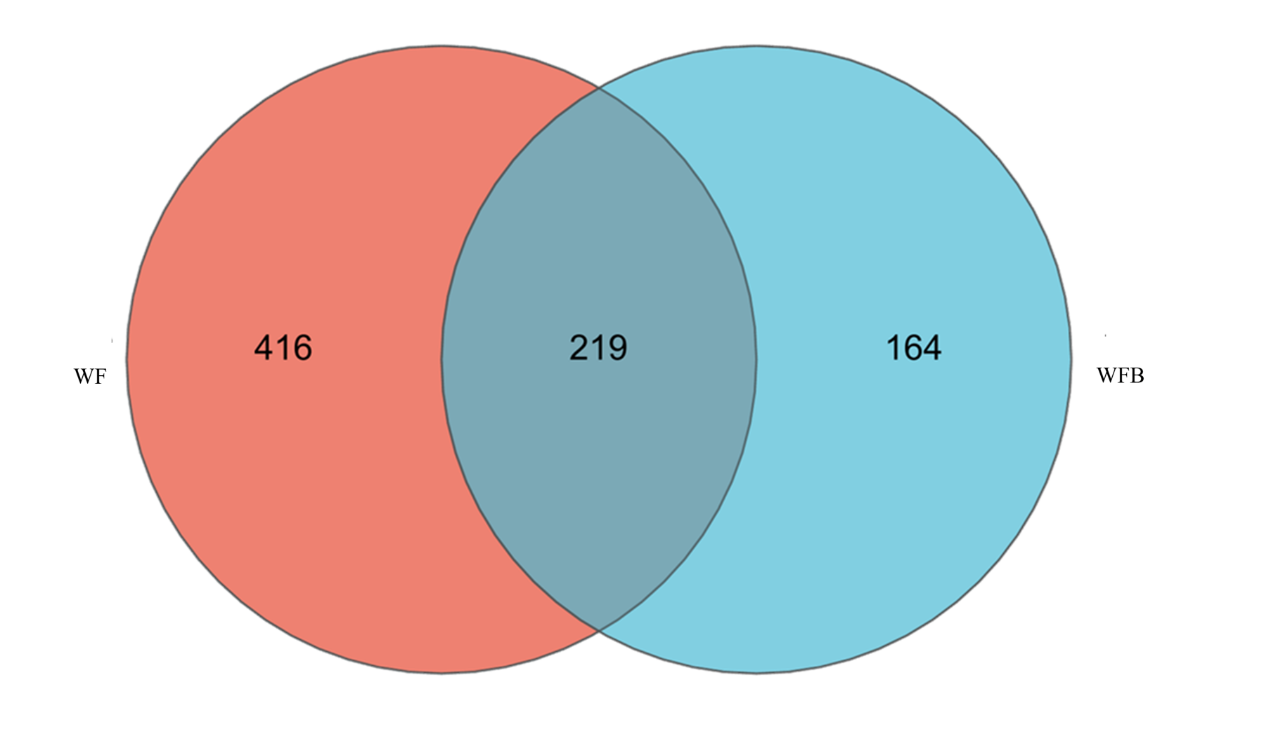


**Supplementary Figure Legends**

**Supplementary Figure 1.** The glucose standard curve. The horizontal coordinate is the glucose concentration, and the vertical coordinate is the absorbance of the solution at OD_540_.

**Supplementary Figure 2.** Venn diagram of species composition. WF, wheat bran with feces from DLY pigs. WFB, wheat bran with feces from DLY pigs and *Bacillus altitudinis* strain Z-99.
